# Predictors of Mortality among Adult Antiretroviral Therapy Users in Southeastern Ethiopia: Retrospective Cohort Study

**DOI:** 10.1155/2015/148769

**Published:** 2015-03-04

**Authors:** Tesfaye Setegn, Abulie Takele, Tesfaye Gizaw, Dabere Nigatu, Demewoz Haile

**Affiliations:** ^1^Department of Reproductive Health, School of Public Health, College of Medicine and Health Sciences, Bahir Dar University, Ethiopia; ^2^Department of Nursing, College of Medicine and Health Sciences, Madawalabu University, P.O. Box 302, 1000 Bale Goba, Ethiopia; ^3^Department of Medicine, College of Medicine and Health Sciences, Madawalabu University, P.O. Box 302, 1000 Bale Goba, Ethiopia; ^4^Department of Public Health, College of Medicine and Health Sciences, Madawalabu University, P.O. Box 302, 1000 Bale Goba, Ethiopia

## Abstract

*Background*. Although efforts have been made to reduce AIDS-related mortality by providing antiretroviral therapy (ART) services, still people are dying while they are on treatment due to several factors. This study aimed to investigate the predictors of mortality among adult antiretroviral therapy (ART) users in Goba Hospital, Southeast Ethiopia. *Methods*. The medical records of 2036 ART users who enrolled at Goba Hospital between 2007 and 2012 were reviewed and sociodemographic, clinical, and ART-related data were collected. Multivariable Cox proportional hazards regression model was used to measure risk of death and identify the independent predictors of mortality. *Results*. The overall mortality incidence rate was 20.3 deaths per 1000 person-years. Male, bedridden, overweight/obese, and HIV clients infected with TB and other infectious diseases had higher odds of death compared with their respective counterparts. On the other hand, ART clients with primary and secondary educational level and early and less advanced WHO clinical stage had lower odds of death compared to their counterparts. *Conclusion*. The overall mortality incidence rate was high and majority of the death had occurred in the first year of ART initiation. Intensifying and strengthening early ART initiation, improving nutritional status, prevention and control of TB, and other opportunistic infections are recommended interventions.

## 1. Introduction

Antiretroviral therapy (ART) has drastically improved the survival of patients who suffer from HIV/AIDS [1]. The primary goals of ART are as follows: maximal and durable suppression of viral replication, restoration of immunologic function, reduction of HIV-related morbidity and mortality, improvement of quality of life, and prolonging survival [2, 3]. Global commitment to scale up HIV treatment in resource-limited settings is bearing fruit. The fast growth in ART coverage represents one of the great public health success stories in recent history of HIV care that lead to reduction of mortality and improvement of quality of life of people living with HIV/AIDS (PLWHA) [1, 3].

The government of Ethiopia has been working on the scaling up of ART for all people to reduce AIDS-related morbidity and mortality. Ethiopian Ministry of Health (MOH) introduced ART since 2003 on subsidized, fee-based scheme and has developed national guidelines on the use of ART. Since 2005, ART became freely available. Further, ART service was decentralized to health centers in 2006, which marked the rapid scale-up phase in the history of the Ethiopian ART program. In addition to adopting the World Health organization (WHO) recommended public health approach, Ethiopia used innovative models such as a nationwide campaign to achieve national targets both for ART and HIV testing and counseling [4–6]. The recent government report indicated that there were 274,805 patients (69% ART coverage) who were alive and on ART by mid-2012 [7]. ART in Ethiopia included Stavudine (D4T), Lamivudine (3TC), Nevirapine (NVP), Zidovudine (AZT), 3TC-NVP, D4T- 3TC-Efavirenz (EFV), and AZT-3TC-EFV prescribed to 46%, 18%, 23%, and 13%, respectively [8].

Despite the scale-up of ART, early mortality is a major challenge. High rates of early mortality were reported from a number of Sub-Saharan African ART programs [9–11]. Age, sex, educational status, place of residence, WHO clinical stage, CD4, Hemoglobin, nutrition, functional status, and opportunistic infections have been associated with mortality among clients on ART [12–15].

However, those factors which contribute to the deaths of HIV-infected patients while on ART are not well explored particularly in Southeast Ethiopia. Therefore, this study aimed to investigate the predictors of mortality among adult ART users in Goba Hospital, Southeast Ethiopia.

## 2. Methods

### 2.1. Study Setting and Period

The study was conducted in Goba Hospital, which is one of the public Hospitals in Bale Zone, located in Goba town 444 kilometers Southeast of Addis Ababa. The hospital has four major departments and one ART clinic. The ART clinic was established in 2005/6 after the Ethiopian government launched free ART service. Currently the ART clinic is providing basic HIV care and treatment. The data collection was conducted from March to April 2013.

### 2.2. Study Design and Sampling Procedure

A retrospective cohort study was conducted on adult ART users. Both ART experienced and ART naïve clients who started treatment in the time frame from 2007 to 2012 (5 years retrospective follow-up) were included in the study.

Medical records of adults (>15 years old) initiating ART between 2007 and 2012 were examined.

### 2.3. Data Collection and Quality Control

Data were extracted using the Federal Ministry of Health (FMOH) HIV care/ART follow-up format as a data extraction tool. The data were extracted by a trained hospital ART service data clerk. The data extraction format includes sociodemographic characteristics (age, sex, marital status, educational level, and occupational status), functional status, duration on ART (measured in months), nutritional status (measured by BMI), WHO clinical staging (from stage I to stage IV), and clinical and laboratory markers. Functional status (bedridden, ambulatory, and working) was recorded from the patients' card and measured based on the status of patient at initiation of ART. The data collection/extraction process was supervised by the investigators. All completed data were examined for clarity and consistency on daily basis before analysis.

### 2.4. Data Analysis

Data were checked for completeness and inconsistencies, coded, entered, cleaned, and analyzed using SPSS for Windows version 20.0 (IBM SPSS Statistics, IBM Corp., New York). Descriptive statistics such as median, interquartile range (IQR), and mean and standard deviation (±SD) were used to summarize the characteristics of the cohort. Person time (years) contribution of each study participant was calculated by comparing ART initiation time and death as an outcome variable. All AIDS-related deaths (which might include deaths due to opportunistic infections secondary to poor adherence to both ART regimen and prophylactic antibiotics) were recorded. ART clients who were lost to follow-up, transferred out, and dropped out were considered as censored.

The Kaplan Meier (KM) curve with Log Rank test was used to describe the survival time of ART patients based on initial CD4 count and WHO clinical stage at initiation of ART. Bivariate and multivariable Cox proportional hazards regression models were used to identify predictors of mortality. Those variables found statistically significant in the bivariate analysis (*P* < 0.05) were entered into multivariable Cox proportional hazards regression model to identify the independent predictors (adjusted for potential confounders) of mortality and estimate the adjusted hazard ratios (AHR). Both crude and adjusted hazard ratios (HRs) with 95% confidence intervals were reported. Variables which were statistically significant at *P* value <0.05 were concluded as predictors of mortality among ART clients.

### 2.5. Concepts and Definitions of Variables

Duration of treatment and follow-up was time (in months) after initiation of ART. Clinical stages (I–IV) were defined based on the WHO classification for AIDS clients. Mortality/death was defined as any recorded AIDS-related deaths including deaths due to opportunistic infections secondary to delayed ART initiation and poor adherence to both ART regimen and prophylactic antibiotics. In this study a client was considered as lost to follow-up if he/she had not been seen for ≥1 month but <3 months while dropped out clients were those lost to follow-up for >3 months. A client who is transferred to another health facility for care was considered as transferred out. Therefore, all ART clients who were lost to follow-up, transferred out, and dropped out were considered as censored. On the other hand, the concept of functional status of clients was labeled as follows: working, to refer to clients who are able to perform usual work in or out of the house; ambulatory, to refer to clients able to perform activities of daily living; and bedridden, designated to clients who are not able to perform activities of daily living.

*Ethical Consideration.* Ethical clearance was obtained from the Institutional Research Ethics Review Committee, Research and Community Service Directorate Office of Madawalabu University. Letter of permission was obtained from administrative body of Goba Hospital. All information collected from clients' cards was kept anonymous and confidential.

## 3. Results

### 3.1. Characteristics of ART Clients

In this cohort study a total of 2036 ART clients were included. More than half 1132 (55.6%) were females and 904 (44.4%) were males. Four hundred twelve (20.2%) of the ART clients had no formal education. The median (IQR) age at ART initiation was 35 years (IQR = 29–38). One thousand seven hundred six (57.8%) and 1197 (58.8%) of ART clients were married and orthodox Christian, respectively (Table 1).

About 1646 (80.8%) of the clients were at working functional status during ART initiation. One thousand one hundred ninety-six (58.7%) of clients initiated ART at WHO clinical stage III. The median baseline CD4 count was 121 per millimeter cube of blood (IQR = 64–169). The median baseline hemoglobin (HgB) was 13.6 mg/dl (IQR = 11 mg/dl–14.6 mg/dl). Three hundred eighty-three (43.3%) of the clients were anemic at initiation of ART (Table 2).

In this study, out of the total of ART clients, 27.3% of them have been on ART for more than 60 months. Nearly half 1101 (54.1%) were alive and on follow-up while 341 (16.7%) and 474 (23.3%) were lost/dropped out and transferred out, respectively.

**Figure 1:**
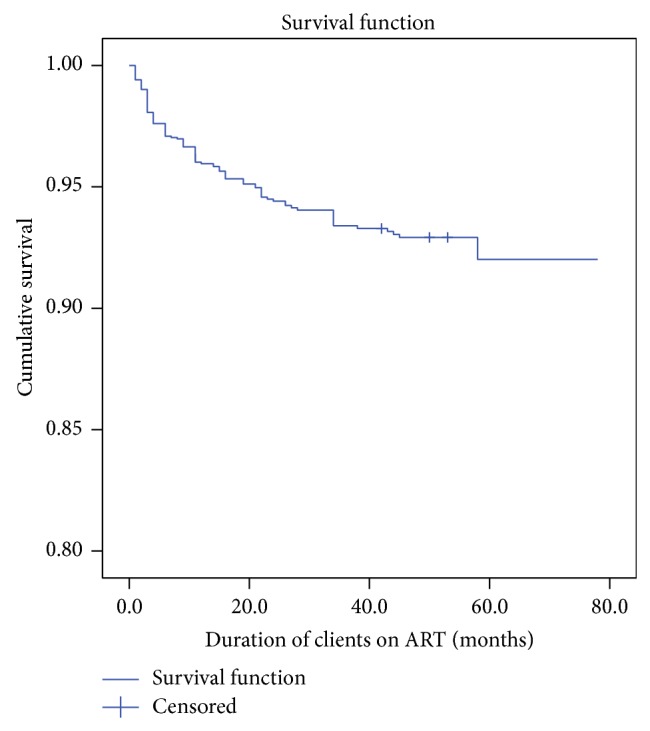
Survival among a cohort of ART clients, Goba Hospital, Southeast Ethiopia, 2013.

There were 120 deaths in 5,912 person-years of retrospective follow-up. The overall mortality incidence rate was 20.3 deaths per 1000 person-years. Of the total 120 deaths, majority of 78 (65.0%) occurred in the first year (<12 months) of ART initiation while twenty-two (18.3%) had died after they have been followed up for 12–24 months. The overall mean (95% CI) survival time was 34.9 (95% CI: 33.8–35.9) months after ART initiation (Figure 1).

**Figure 2:**
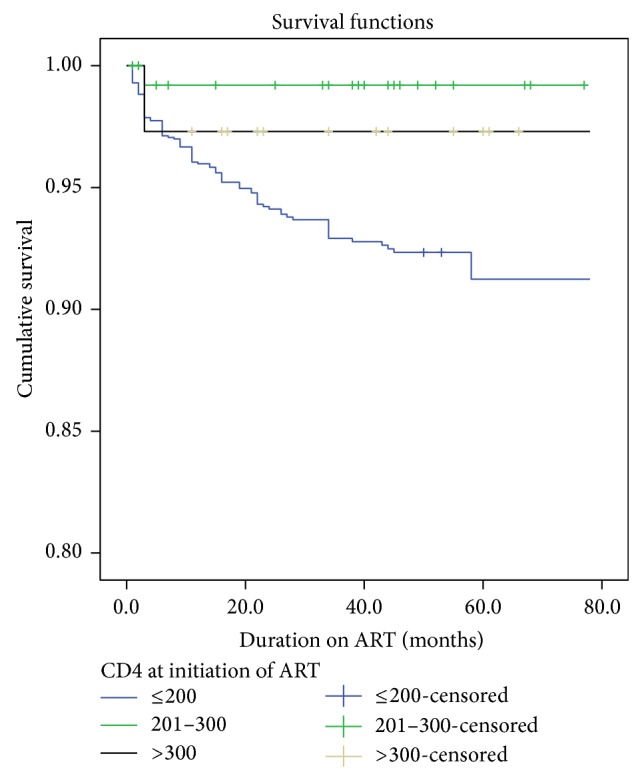
Survival function stratified according to initial recorded CD4 count among a cohort of ART clients, Goba Hospital, Southeast Ethiopia, 2013.

The equality of survival distribution (based on Log Rank test) between different categories of initial CD4 count showed a statistically significant difference in survival of clients based on different categories of initial CD4 (*χ* ^2^ = 13.73, Df = 2; *P*  value < 0.001) (Figure 2).

**Figure 3:**
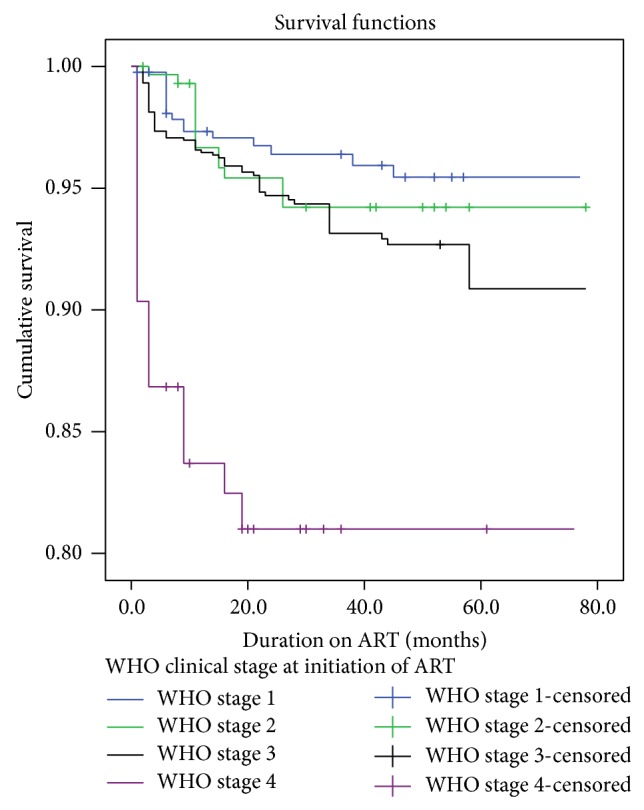
Survival function stratified according to initial WHO clinical stage among a cohort of ART clients; Goba hospital, Southeast Ethiopia; 2013.

Similarly, the survival distribution of ART clients based on initial WHO clinical stage showed a statistically significant difference on Log Rank test (*χ* ^2^ = 40.01, Df = 3; *P*  value < 0.001) (Figure 3).

### 3.2. Predictors of Mortality

In our study, multivariable Cox proportional hazards regression analysis showed that male ART clients, ambulatory clients, TB coinfected clients, individuals with baseline BMI ≥ 25 kg/m^2^, and clients who had opportunistic infection had higher odds of early death compared with their respective counterparts. On the other hand, higher educational status was associated with lower odds of early mortality compared to clients with no formal education. Similarly clients who had better baseline WHO clinical stages (I–III) had lower odds of early mortality compared to clients with baseline WHO clinical stage IV.

Male ART clients were 2.6 times more likely to experience early mortality while on ART treatment compared to females (AHR = 2.67; 95% CI: 1.74–4.10). Similarly, bedridden clients were 4.4 times more likely to die early as compared to clients with working functional status (AHR = 4.4; 95% CI: 1.55–12.36). Those ART clients who are TB coinfected at ART initiation were 4.5 times more likely to experience early mortality (AHR = 4.51; 95% CI: 2.86–7.11). On the contrary, those ART clients with primary and secondary education had 72% (AHR = 0.28; 95% CI: 0.11–0.70) and 66% (AHR = 0.34; 95% CI: 0.154–0.728) lower odds of death as compared to clients with no formal education, respectively. Compared to WHO clinical stage IV, being at lower WHO clinical stages (I–III) was found to be protective of early mortality for clients on ART. ART clients with WHO clinical stages I, II, and III had 84%, 66%, and 76% lower odds of early mortality compared with clients with WHO clinical stage IV. However, age of clients, CD4 counts, and anemia status were not significantly associated with odds of death among ART clients (Table 3).

## 4. Discussion

In this study, there were 120 deaths among ART clients per 5,912 person-years of observation making the overall mortality rate 20.3 deaths per 1,000 person-years. This finding is similar to the finding from Eastern Ethiopia, which reported an overall mortality incidence rate of 2.03/100 person-years [12], whereas studies conducted in rural public hospitals of Southern Nations, Nationalities, and People Regional (SNNPR) state of Ethiopia [16] and Arbaminch hospital [17] have reported higher mortality incidence rates which were 74.5 deaths per 1,000 and 15.4 deaths per 100 person-years, respectively.

Our study showed that majority (65%) of the deaths occurred in the first year of ART initiation which was a similar finding with the study conducted in Arbaminch Hospital, Southern Ethiopia [10]. A study from Uganda showed that mortality rate in the first year of ART initiation was 12 deaths per 100 person-years [18]. Early and higher mortality of ART clients in this study was supported by different studies that revealed high early mortality in ART cohort that has been evidenced specially in resource-limited settings [11, 13, 19–21]. Deaths that occurred within the first early months of therapy might result from treatment failure, severe drug toxicity, and regimen changes [22].

Regarding the factors associated with mortality of ART clients, the Cox regression analysis showed that male ART clients were more likely to die compared to female ART clients. This finding is similar to several studies [11, 14, 16, 18, 21, 23–26]. The possible reason for higher risk of mortality among male ART clients might be due to the observed significant difference in the proportion of baseline CD4 count (CD4 at initiation ART) <200/mm^3^ (*P* < 0.001) between males and females (90.5% versus 81.6%). The other possible explanation for higher risk of mortality in males might be due to the males' behavioral factors like substance use [27–31].

In our study, those ART clients with higher educational status (primary and secondary education) had lower odds of death compared to those ART clients with no formal education. Many studies documented that higher educational status was associated with lower risk of mortality among ART users [15, 32, 33]. Likewise, those bedridden clients at initiation of ART were more likely to die compared to working functional status clients. In this regard, studies from Nepal and Ethiopia reported supporting evidences that poor baseline performance scale (being bedridden) was a predictor of mortality during ART care [12, 21, 34]. In the same way, WHO clinical stages (I–III) were associated with lower odds of death as compared to clients with WHO clinical stage IV. This indicated that advanced baseline WHO clinical stage was statistically significant clinical predictor of mortality as comparably documented by different studies [11, 12, 14, 16, 25, 35, 36]. In our study, apart from advanced WHO clinical stage, TB coinfection at ART initiation was significantly associated with higher odds of mortality among ART clients as analogously reported by various studies [14, 18, 25, 35, 37]. ART clients who had BMI ≥ 25 Kg/m^2^ had higher odds of mortality compared with clients with normal BMI (18.5–24.9 Kg/m^2^) category. Higher odds of death among obese ART clients might be associated with other chronic diseases related to overweight and obesity such as hypertension, diabetes mellitus, and others. Although different studies documented that anemia is a statistically significant predictor of mortality of ART clients [13–15, 25, 34–36], this study did not find statistically significant association between anemia and mortality of ART clients.

This study should be interpreted in light of its strengths and limitations. Relatively larger sample size from long period of retrospective follow-up and the use of both clinical and nonclinical data were the strengths of this study. As a limitation, this study used routinely collected health care data which might have been introduced under estimation of mortality due to many unreported/home deaths. On the other hand, since the data were secondary data, authors could not ascertain that all recorded deaths were AIDS-related deaths (which might include deaths due to opportunistic infections secondary to poor adherence to both ART regimen and prophylactic antibiotics). The analysis was restricted only on the variables recorded on the clients' card. Therefore, interpretation and conclusion of finding should take these limitations into account.

## 5. Conclusion

The overall mortality incidence rate was 20.3 deaths per 1000 person-years. The presence of TB coinfection, WHO clinical stage, being bedridden, educational status, presence of opportunistic infections, and being overweight and obese were factors associated with mortality among ART clients. Initiation of ART at early WHO clinical stage, improving nutritional status, prevention and control of TB, and other opportunistic infections were the recalled recommendations to decrease AIDS-related morality.

## Figures and Tables

**Table 1:** Sociodemographic characteristics of ART clients, Goba Hospital, Southeast Ethiopia, 2013.

Sociodemographic variables	Frequency	Percent
Age (years)		
15–24	179	8.8
25–29	393	19.3
30–34	419	20.6
35–39	572	28.1
40–44	220	10.8
≥45	253	12.4
Sex of participants		
Male	904	44.4
Female	1132	55.6
Educational level		
No education	412	20.2
Primary	689	33.8
Secondary	801	39.3
Tertiary	134	6.6
Occupational status		
Merchant	311	15.3
Day laborer	302	14.8
Housewife	535	26.3
Jobless	257	12.6
Farmer	231	11.3
Government employee	259	12.7
Other^*^	141	6.9
Marital status		
Never married	325	16.0
Married	1176	57.8
Separated	138	6.8
Divorced	289	14.2
Widowed	108	5.3
Religion		
Orthodox	1197	58.8
Muslim	670	32.9
Protestant	139	6.8
Others^®^	30	1.5

^*^Students, construction workers, drivers, and servants, ^®^Jehovah, wakefeta.

**Table 2:** Clinical characteristics of ART clients, Goba Hospital, Southeast Ethiopia, 2013.

Baseline^*^ characteristics	Number	Percent
Baseline functional status		
Working	1646	80.8
Ambulatory	276	13.6
Bedridden	114	5.6
WHO stage at ART initiation		
WHO stage I	422	20.7
WHO stage II	304	14.9
WHO stage III	1196	58.7
WHO stage IV	114	5.6
Baseline CD4 count		
≤200	1705	85.5
201–300	252	12.6
>300	37	1.9
Median CD4 (mm^3^) (IQR)	121.0 (169.0–64.0) mm^3^	
Baseline HgB level		
Anemic	383	43.3
Nonanemic	501	56.7
Median HgB (mg/dL) (IQR)	13.6 mg/dL (11 mg/dL–14.6 mg/dL)	
Patient screened for TB		
Yes	1940	95.3
No	96	4.7
TB coinfection at initiation of ART		
Positive	285	14.7
Negative	1655	85.3
Baseline BMI		
<18.5	11	1.3
18.5–24.9	756	88.6
≥25.0	86	10.1
Median weight (Kg) (IQR)	50.0 Kg (57 Kg–45 Kg)	

^*^Baseline indicates measurements at initiation of ART.

**Table 3:** Cox regression model: Selected sociodemographic and clinical factors associated with mortality of ART clients in Goba Hospital, Southeast Ethiopia, 2013.

Variables	Death as an outcome	
	Unadjusted HR (95% CI)	Adjusted HR (95% CI)
Sex of participants		
Male	3.60 (2.40–5.30)^*^	2.67 (1.74–4.10)^**^
Female	1.0	1
Educational level		
No formal education	1.0	1.0
Primary	0.34 (0.13–0.84)^*^	0.28 (0.11–0.70)^**^
Secondary	0.65 (0.31–1.39)	0.34 (0.15–0.73)^**^
Tertiary	1.53 (0.77–3.05)	0.71 (0.34–1.46)
Functional status		
Working	1.0	
Ambulatory	1.11 (0.41–3.05)	1.62 (0.55–4.75)
Bedridden	4.99 (1.80–13.88)^*^	4.38 (1.55–12.36)^**^
Baseline WHO stage		
WHO stage 1	0.17 (0.09–0.33)^*^	0.16 (0.08–0.33)^**^
WHO stage 2	0.22 (0.11–0.44)^*^	0.34 (0.16–0.73)^**^
WHO stage 3	0.30 (0.18–0.49)^*^	0.24 (0.13–0.43)^**^
WHO stage 4	1.0	
Baseline CD4 count		
≤200	2.5 (0.3–17.6)	
201–300	0.3 (0.03–3.20)	—
>300	1.0	
TB coinfection		
Positive	4.7 (3.2–6.8)^*^	4.51 (2.86–7.11)^**^
Negative	1.0	1
Anemia status		
Anemic	0.25 (0.04–1.52)	—
Nonanemic	1.0	
Baseline BMI (kg/m^2^)		
<18.5	1.83 (1.20–2.77)^*^	1.20 (0.77–1.87)
18.5–24.99	1.0	
≥25.0	3.19 (1.61–6.36)^*^	3.71 (1.81–7.59)^**^
Opportunistic infection		
Yes	6.27 (4.12–9.54)^*^	2.51 (1.46–4.31)^**^
No	1.0	
Age group		
15–30 years	1.47 (0.88–2.45)	
31–40 years	0.84 (0.49–1.42)	—
≥41 years	1.0	

HR: hazard ratio; ^*^statistically significant at P < 0.05 at the unadjusted HR model; ^**^statistically significant at P < 0.05 at the adjusted HR model (both 2 tailed).

## Abbreviations

AIDS: Acquired immunodeficiency syndrome
ART: Antiretroviral therapy
FDRE: Federal Democratic Republic of Ethiopia
HgB: Hemoglobin
HIV: Human immunodeficiency virus
IQR: Interquartile range
MOH: Ministry of Health
PLWHA: People living with HIV/AIDS
RCSDO: Research and Community Service Directorate Office
TB: Tuberculosis
WHO: World Health Organization.
